# Cellular senescence and senolytic therapy in traumatic brain injury

**DOI:** 10.3389/fnins.2026.1862748

**Published:** 2026-08-05

**Authors:** Darrell W. Brann, Harminder D Singh, Jing Wang, Yujiao Lu, Christopher Carr, Krishnan M. Dhandapani

**Affiliations:** 1 Department of Neurosurgery, Medical College of Georgia, Augusta University, Augusta, GA, United States; 2 Department of Neurology, Medical College of Georgia, Augusta University, Augusta, GA, United States

**Keywords:** cellular senescence, cGAS-STING, inflammation, senescence associated secretory phenotype, senescent cell, senolytic, traumatic brain injury

## Abstract

Traumatic brain injury (TBI) is a major cause of death and long-term disability in the United States. The initial primary injury in TBI is followed by a secondary injury cascade of molecular events, which can persist for years, and contributes to neuroinflammation, neurodegeneration, and long-term functional deficits after TBI. In this review, we will discuss evidence that cellular senescence in TBI, where damaged cells enter a state of permanent cell-cycle arrest and release pro-inflammatory factors, is a component of the secondary injury cascade, which contributes to both chronic neuroinflammation and long-term neurodegeneration after TBI. There is now abundant evidence that a single moderate to severe TBI or repeated mild TBI leads to DNA damage and oxidative stress, which triggers cellular senescence in the injured brain. The induction of cellular senescence leads to production of a cocktail of pro-inflammatory cytokines, chemokines, and matrix remodeling proteases, collectively termed the senescence associated secretory phenotype (SASP). While the SASP may be beneficial acutely in certain situations, chronically it has been suggested to promote a pro-inflammatory and pro-neurodegenerative environment. Additional work using both global and cell-specific knockout animal models indicates that the cGAS-STING signaling pathway helps connect cellular damage to the SASP, as it detects cytosolic DNA in damaged cells and regulates SASP production. Finally, we will discuss future directions for the field, and review evidence that therapeutically targeting of senescent cells through administration of senolytic drugs in animal models leads to attenuated neuroinflammation and neurodegeneration in the injured brain, and enhances functional outcome after TBI.

## Cellular senescence

Cellular senescence is a state in which cells permanently stop dividing and enter into a terminal non-reversible cell cycle arrest, often triggered by stress, damage or aging (Campisi and D'adda Di Fagagna, 2007; Tchkonia et al., 2013). Both beneficial and detrimental effects have been implicated for cellular senescence following tissue injury (Huang et al., 2022; Baker et al., 2023; Wang et al., 2024). Proposed beneficial effects include: (1) accelerating wound closure and enhancing tissue repair, (2) limiting proliferation of damaged cells to prevent tumor development, and (3) helping drive tissue patterning and morphogenesis in embryonic development. Conversely, proposed detrimental effects of cellular senescence include: (1) chronic inflammation, (2) tissue degradation, (3) stem cell depletion, and (4) aging and disease progression. It seems that duration, tissue microenvironment, and clearance by the immune system are key factors that determine whether cellular senescence is beneficial or detrimental (Baker et al., 2023; Wang et al., 2024). Generally, when active for brief periods, cellular senescence appears to act as a protective, adaptive mechanism. However, if senescent cells persist and are not cleared out, then cellular senescence can often be detrimental. In this narrative review, we will discuss evidence for the induction of cellular senescence following traumatic brain injury (TBI), its potential contribution to TBI pathology, and whether clearance of senescent cells from the injured brain could potentially have therapeutic benefit.

**Figure 1:**
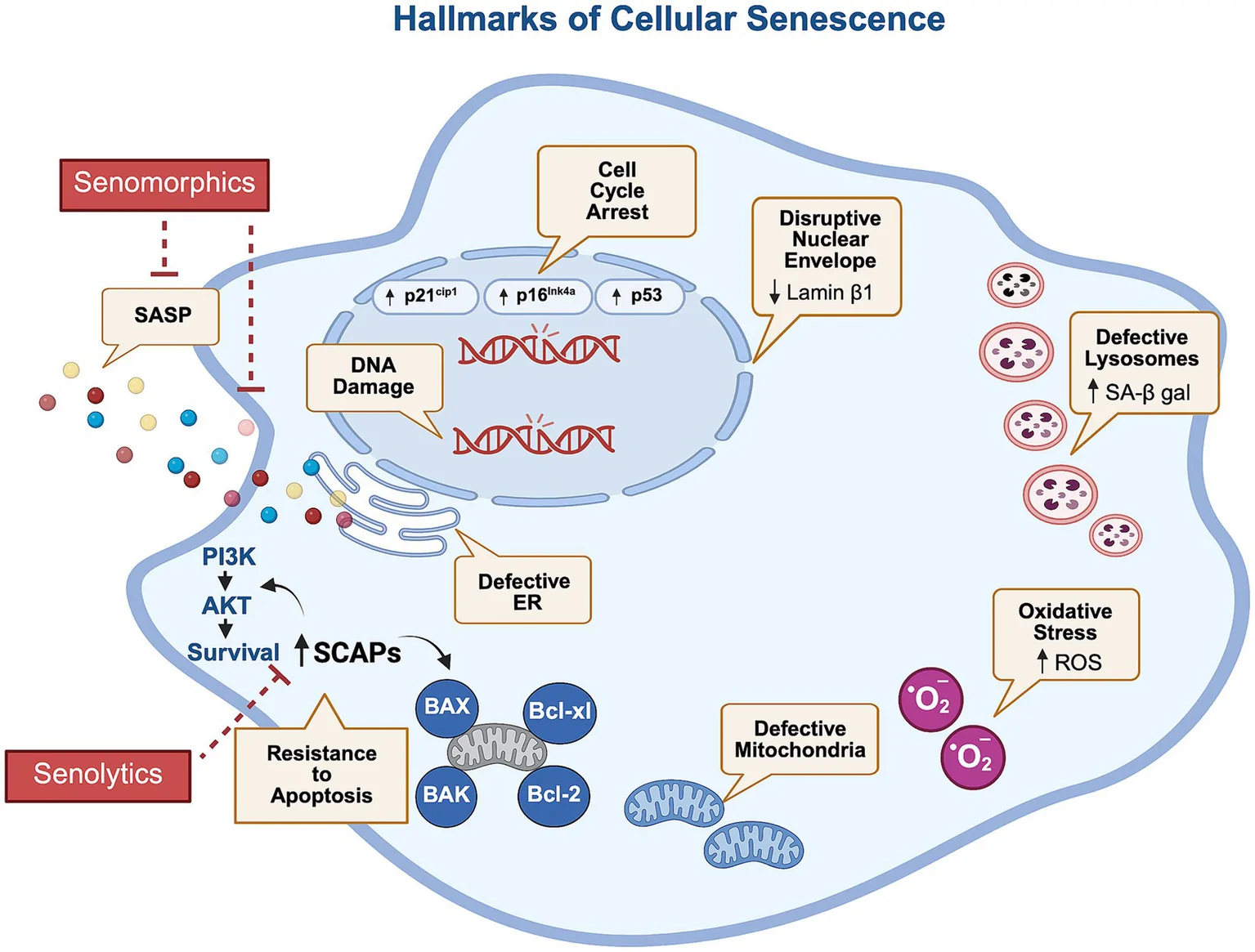
Hallmarks of cellular senescence. The major hallmarks of cellular senescence are depicted in this illustration, which includes cell cycle arrest through increase of cell cycle inhibitor factors, disruption of the nuclear envelope with loss of nuclear lamin β1, genotoxic stress as reflected by DNA damage, defective ER and mitochondria, oxidative stress as reflected by increased ROS, increased resistance to apoptosis as reflected by senescent cell anti-apoptotic pathways (SCAPs) such as PI3K/AKT pro-survival pathway and Bcl-2/Bax anti-apoptotic pathway, which can be targeted by senolytic drugs to ablate senescent cells, defective lysosomes with increased SA-β galactosidase (SA-β gal), and production of proinflammatory factors, termed the senescence-associated secretory phenotype (SASP), which can be inhibited by senomorphic drugs. Created in https://BioRender.com.

Senescent cells exhibit many hallmarks by which they can be identified (Figure 1). These include (1) increased cell cycle arrest and over-expression of the cell cycle inhibitors, p21^Cip1/Waf1^ (p21), p16^Ink4a^ (p16), p19^Ink4d^ (p19), (2) DNA damage, (3) decreased nuclear lamin β1 expression and disruption of the nuclear envelop, (4) lysosome dysfunction with increased senescence-associated β-galactosidase (SA-β-gal) expression, (5) mitochondrial and ER dysfunction, and (6) increased reactive oxygen species (ROS) (Figure 1) (Zhang et al., 2022). Furthermore, another key hallmark is that senescent cells become resistant to cell death by up-regulating senescent cell anti-apoptotic pathways (SCAPs), such as anti-apoptotic BCL-2 family genes and the cell survival PI3K/AKT signaling pathway, that stabilize the anti-apoptotic proteins (Kirkland and Tchkonia, 2020; Zhang et al., 2021; Saliev and Singh, 2025). Therapeutically, SCAPs are a prime target for senolytic drugs, which induce apoptosis in senescent cells by disabling the anti-apoptotic/pro-survival pathways (Kirkland and Tchkonia, 2020; Zhang et al., 2021). Another key hallmark of senescent cells is that they can contribute to inflammation in tissues through the secretion of pro-inflammatory factors, such as interleukin-1 beta (IL-1β), interleukins 6, 7 and 8 (IL-6, IL-7, IL-8), interferons, tumor necrosis factor-alpha (TNF-α), chemokines, proteases and growth factors (Kirkland and Tchkonia, 2020). The term “senescence associated secretory phenotype (SASP)” has been coined to describe this cocktail of proinflammatory factors released by senescent cells (Figure 1) (Coppe et al., 2008; Wang et al., 2024). Several signaling pathways, such as NF-κB, mTOR and JAK/STAT, are responsible for SASP production (Kirkland and Tchkonia, 2020; Zhang et al., 2021; Saliev and Singh, 2025). Furthermore, SASP production can be inhibited by a class of drugs called senomorphics, which target these signaling pathways (Figure 1) (Kirkland and Tchkonia, 2020; Zhang et al., 2021; Saliev and Singh, 2025). Once produced, SASP factors can exert paracrine effects to influence neighboring cells and tissues, often amplifying senescence signals or promoting tissue repair in the short term. Conversely, chronic SASP induction has been suggested to help drive aging and disease by contributing to persistent inflammation and exacerbating tissue dysfunction, (Wang et al., 2024). Senescent cells occur in many tissues of the body and increase with aging (Baker and Petersen, 2018). Furthermore, research in animals has shown that pharmaceutical or genetic ablation of senescent cells extends the life span and health span (Baker et al., 2011; Baker et al., 2016; Xu et al., 2018). In the brain, cellular senescence has been shown to occur in both astrocytes and microglia and contribute to aging and neurodegenerative disorders (Baker and Petersen, 2018; Kritsilis et al., 2018). For instance, senescent glia cells have been described in individuals suffering from Alzheimer’s disease (AD), Parkinson’s disease (PD), and stroke (Streit et al., 2009; Bhat et al., 2012; Lim et al., 2021; Lu et al., 2023; Carling et al., 2024; Rachmian et al., 2024; Rim et al., 2024), as well as in individuals who had suffered a traumatic brain injury (TBI) (Schwab et al., 2019a). Since there is no single gold standard for detection of cellular senescence, investigators typically use a combination of markers to confirm cellular senescence such as measurement/detection of SA-β-gal, p16, p21, p19 cell cycle inhibitors, SASP factors, DNA damage response markers, and morphology and structure markers such as enlarged, flattened, irregular cell shape, and reduced nuclear lamin β1.

## TBI and cellular senescence

TBI is one of the leading causes of morbidity and long-term disability in the United States (Thurman et al., 1999). TBI is induced by an acute trauma, such as a blow or jolt to the head, and it is estimated that over 11 million people in the United States are living with a disability that resulted from a TBI (Schneider et al., 2021). Although induced acutely, there is increasing evidence that TBI is a chronic and progressive disorder (Simon et al., 2017). For instance, significant injury-related disabilities such as anxiety, cognitive impairment, and/or depression have been reported in hospitalized TBI survivors 1 year after injury (Schneider et al., 2021). Furthermore, white matter damage and neuroinflammatory events have been shown to persist in the human brain many years after TBI (Ramlackhansingh et al., 2011; Johnson et al., 2013). Based on these findings, and others, it has been proposed that over time, neuroinflammatory signals cause spread of the initial injury to nearby healthy regions – leading to amplification of the original focal tissue injury (Loane et al., 2014; Lozano et al., 2015).

**Figure 2:**
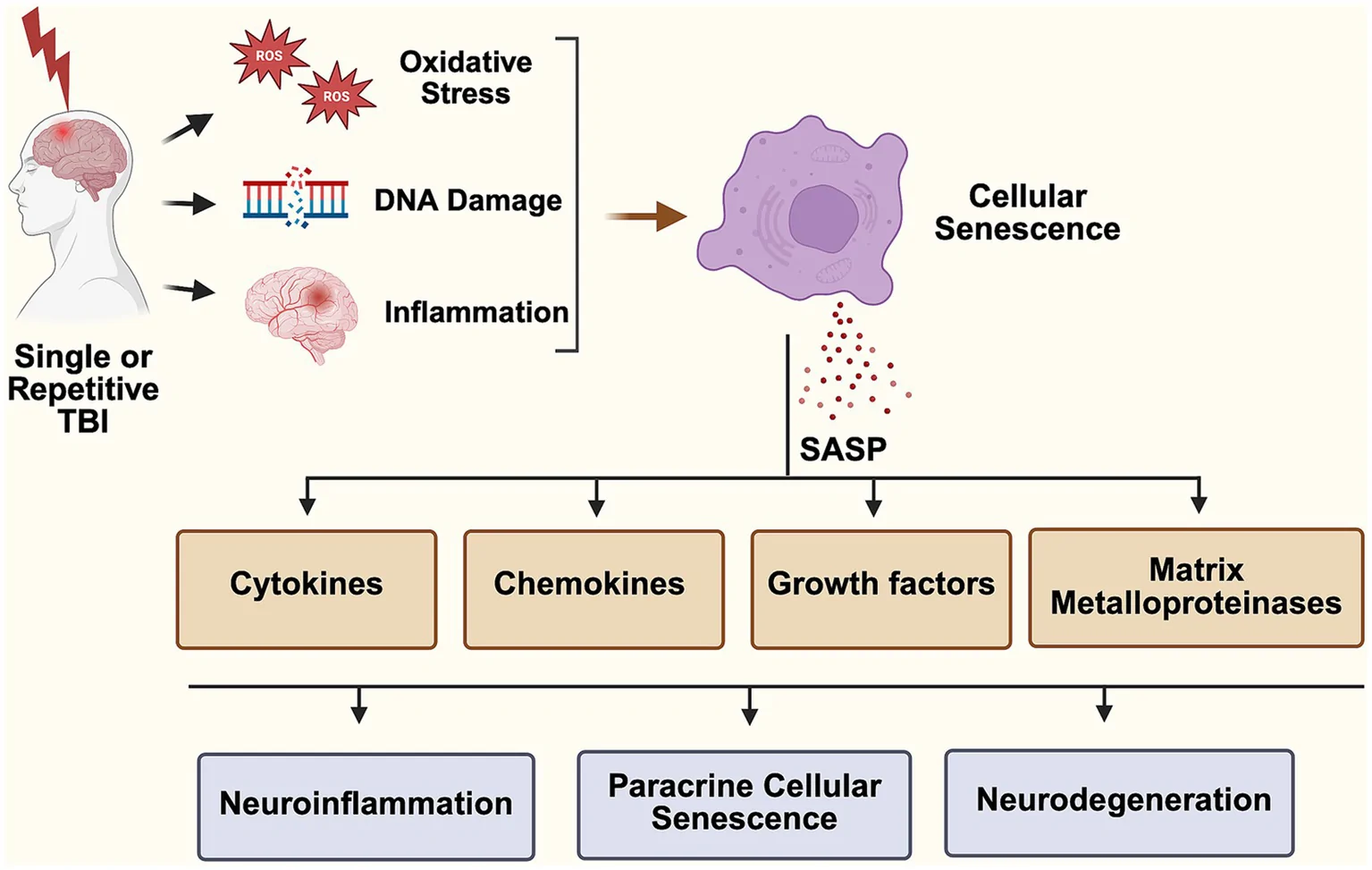
Proposed mechanisms of cellular senescence following single or repetitive TBI. A single moderate or severe TBI or repetitive mild TBI produces oxidative stress, inflammation, and DNA damage in the brain. These processes contribute toward the development of cellular senescence and SASP, which potentiate neuroinflammation, spread of cellular senescence in a paracrine manner to nearby healthy cells, and progressive neurodegeneration. Created in https://BioRender.com.

Several studies have provided evidence of the induction of cellular senescence in the brain following either single or repetitive TBI. Figure 2 shows a hypothetical model on how cellular senescence may be induced following TBI, and how chronically it may contribute to neuroinflammation and neurodegeneration. As shown, a single moderate or severe TBI or repetitive mild TBI can lead to oxidative stress, DNA damage, and inflammation, which facilitates the induction of cellular senescence in the brain. The induction of cellular senescence after TBI leads to generation of a pro-inflammatory SASP, which may contribute to chronic neuroinflammation, spread of cellular senescence to adjacent healthy cells, and progressive neurodegeneration (Figure 2). As mentioned previously, in some situations cellular senescence may actually be beneficial, by limiting proliferation of damaged cells and enhancing tissue repair. However, if the senescent cells persist, they could contribute to a proinflammatory environment and detrimental effects long-term after TBI. In one of the first reports on cellular senescence after TBI, Kozlova and Lukanionin (Kozlova and Lukanidin, 2002) found that p16 immunoreactive protein expression was strongly increased in astrocytes in the injured cerebral cortex and in white matter at 4 days, 1 week, and 1 month after a penetrating injury to the female rat brain. Similarly, a study in young male mice (10-weeks old) found that controlled cortical impact (CCI) significantly increased SA-β-gal immunostaining in the area adjacent to the contusion area of the cerebrum at 4 days, 7 days and 14 days after TBI, with the peak observed at 7 days (Tominaga et al., 2019). In addition, p16, p21 and p53 mRNA expressions were also increased in the cerebrum at 4 days, 7 days and 14 days after TBI. Immunostaining further revealed that p16 immunoreactive protein was induced in astrocytes, while p21 was observed in neurons and microglia of the cerebrum on the ipsilateral side at 4 days, 7 days and 14 days after TBI (Tominaga et al., 2019). Cyclin D1- and PCNA-immunostained neurons, astrocytes and microglia were also found in the injured cerebrum after TBI, which the authors suggested indicates that cell cycle activation is initiated soon after TBI, and that cells with activated cell cycle rapidly proceed to cellular senescence after TBI (Tominaga et al., 2019). The study also found that mRNA levels of the pro-apoptotic factor, p53 was increased in the injured cerebrum at 4 days, 7 days and 14 days after TBI, but the cell type(s) where the increase occurred was not indicated, nor whether the increase occurred in senescent or non-senescent cells. However, recent work has shown that senescent cells have mechanisms to inhibit p53 activity to remain viable (Sturmlechner et al., 2022). Likewise, another study found that CCI-induced TBI led to an increased in gene expression of p16, p19 and p53 in the cortex at 5 days post injury in juvenile (4–5 weeks old) male mice (Al-Khateeb et al., 2024). This indicates that cellular senescence may be induced in both the juvenile and adult brain after TBI. Similar to the results observed in the CCI model of TBI, adult male rats exposed to blast TBI exhibited a significant increase in SA-β-gal staining in many brain regions including the cortex and hippocampus at 24 h and 1 month after injury, with 1 month showing the most pronounced increase in SA-β-gal (Arun et al., 2020). Further work also showed that repeated mild TBI in mice increased protein levels for the senescence markers p21 and p16 in the ipsilateral cortex and underlying hippocampus at 1 week after the last mild TBI (Schwab et al., 2022). In contrast to the previously discussed studies, which primarily examined only male animals, this study examined both male and females and found similar changes in each, with the exception that females had lower p16 protein expression after repeated mild TBI than males (Schwab et al., 2022).

Although TBI is an acute injury, it can lead to chronic neuroinflammation and progressive neurodegeneration. In light of this, our group and others examined whether cellular senescence exists long-term (several months) after TBI (Arun et al., 2020; Wang et al., 2023; Exline et al., 2025). In a study from our group, adult male mice were subjected to CCI, and it was shown that gene expression of the senescence markers p16, p21, and IL-1β were significantly increased in the injured cerebral cortex at 4 months after TBI (Wang et al., 2023). Double immunohistochemistry results for the astrocyte marker, GFAP and the senescence markers p16 and p21 revealed that immunoreactive p16 and p21 proteins were significantly induced in astrocytes at 5 weeks and 4 months after TBI in several brain regions, including the cerebral cortex, corpus callosum, dentate gyrus and lateral posterior of thalamus. Induction of immunoreactive p16 protein was also observed in microglia at 4 months after TBI in multiple brain regions (Wang et al., 2023). Likewise, Arun et al. (2020) examined adult male rats exposed to blast TBI and showed a prolonged elevation of SA-β-gal in the geniculate nucleus, hippocampus and ventral thalamic nucleus at 1-year after blast injury. Finally, repeated mild TBI in a rat model also showed a progressive increase in gene expression of the cell senescence markers p16, p21 and p53 in the hippocampus at 5 weeks and 9 weeks post injury (Exline et al., 2025). Collectively, these findings suggest that cellular senescence may exist long-term after TBI, and could potentially contribute to chronic neuroinflammation after TBI. However, since many of the studies on cell senescence and TBI utilized only male animals and examined only 1–2 senescence markers, and only a few provided spatial or cell-specific context, additional studies are needed that emphasize multi-marker validation for cellular senescence, spatial context and cell-type specificity, examination of both male and females, as well as incorporation of transcriptomic and genetic approaches.

## Mechanisms involved in cellular senescence and neuroinflammation after TBI

### DNA damage and senescence-associated secretory phenotype (SASP)

Induction of cellular senescence after TBI has been suggested to be due, at least in part, to DNA damage (Figure 2), which activates the DNA damage response (DDR) and the p53-p21 pathways (Di Micco et al., 2021; Huang et al., 2022). In support of this suggestion, single strand and double strand DNA breaks have been reported in the ipsilateral hemisphere as early as 30 min after moderate TBI in the rat, with peak DNA damage found 6–24 h after TBI (Clark et al., 2001). Postmortem studies in humans also revealed significant DNA damage in astrocytes, oligodendrocytes, and ependymal cells of brains from professional athletes with a history of repeated mild TBI, and reduced expression of DNA repair enzymes (Schwab et al., 2019a). γH2AX, a marker of double stranded DNA damage, has also been found to be increased in the hippocampus, frontal cortex and brainstem in human subjects with a history of mild TBI (Schwab et al., 2019b). In male mice, repeated mild TBI has been shown to induce DNA damage response markers and reduce DNA repair mechanisms in the injured cortex at 24 h to 7 days after TBI (Schwab et al., 2021). The increased DNA damage was associated with increased expression of cellular senescence in the injured cortex at 7 days after TBI in male mice (Schwab et al., 2021). Aging appears to enhance TBI-induced DNA damage and expression of senescence markers, as 12 month old mice showed significantly higher expression of γH2AX, BCL-2, p16, and p21 in microglia at 72 h after CCI, as compared to expression in young (3 month old) mice at 72 h after CCI (Ritzel et al., 2019). However, another study found no age-related differences in DNA damage induction in 3 month old versus 22 month old mice when examined at 24 h after TBI (Barrett et al., 2021). It is not clear why the two studies disagree, but it could be due to the different ages and timepoints that were examined in the studies. Further studies are needed to better clarify this issue.

Following induction of cellular senescence, senescent cells secrete a wide array of proinflammatory factors termed the SASP (Coppe et al., 2008; Wang et al., 2024). The first report of an increase of SASP in the brain after TBI was a study by Schwab et al. (2019a) who found increased gene expression of pro-inflammatory cytokines, chemokines and interleukins in the brains of professional athletes who had experienced repeated mild TBI. Similarly, RNAseq analysis and Nanostring gene expression panel analysis showed significantly increased SASP factors in the brains of mice 1-week after repeated mild TBI (Schwab et al., 2021; Schwab et al., 2022). Thus, it appears that even mild TBI, if repetitive, can induce cellular senescence and SASP in the brain in both humans and mice. In studies by our group, we examined the SASP factors, IL-1β and IL-6, and found their protein expression levels to be significantly increased in the injured male mouse cortex at 18 weeks after CCI (Wang et al., 2023). Thus, SASP induction appears to be a sequela of TBI and can exist in the injured brain both acutely and long-term after TBI. Below, we discuss recent evidence implicating the cyclic GMP-AMP synthase (cGAS) and stimulator of interferon genes (STING) signaling pathway in linking DNA damage to SASP induction and potentially helping mediate neuroinflammation and neurodegeneration after TBI.

### cGAS-STING signaling in TBI

**Figure 3:**
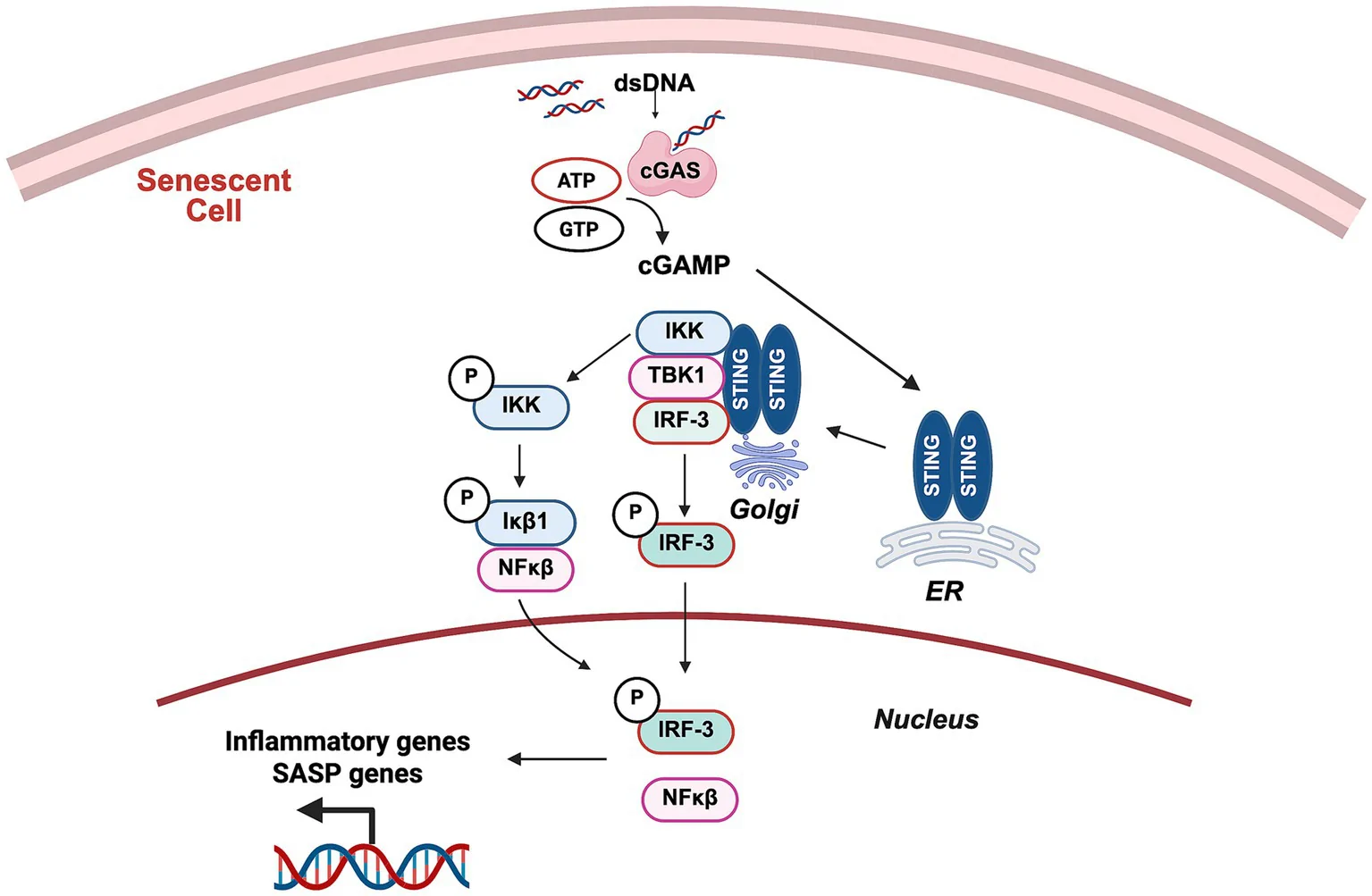
cGAS-STING signaling pathway in senescent cells. Cytosolic cyclic GMP-AMP synthase (cGAS) binds to dsDNA. This leads to activation of cGAS, which catalyzes ATP and GTP into 2′3′-cyclic GMP-AMP (cGAMP). cGAMP binds to and activates the STING protein located on the endoplasmic reticulum (ER), followed by STING translocation to the Golgi apparatus. At the Golgi, STING recruits and activates kinases, including TANK-binding kinase 1 (TBK1) and the IΚK complex (Inhibitor of nuclear factor-B kinase, IκB). The activated IKK complex phosphorylates IκB1α, the inhibitory protein holding NF-κB in the cytoplasm, causing the proteosome to degrade IκB1α, which allows release of NF-κB to translocate to the nucleus, where it induces transcription of inflammatory genes and SASP genes. Simultaneously, STING-activated TBK1 phosphorylates IRF3, which dimerizes and enters the nucleus to further contribute to induction of inflammatory genes and SASP genes. Created in https://BioRender.com.

The cGAS-STING signaling pathway has been implicated as a major pathway sensing cytosolic DNA in damaged cells, leading to SASP induction and neuroinflammation (Gulen et al., 2023). As shown in Figure 3, cGAS is a cytosolic DNA sensor that detects pathogen-derived foreign DNA and DNA released during cellular damage (Decout et al., 2021). DNA binding to cGAS causes production of cyclic GMP-AMP (cGAMP), a second messenger of cGAS, which binds to and activates the adaptor protein, stimulator of interferon genes (STING), which is located in the endoplasmic reticulum. The activated STING oligomerizes and moves to the Golgi apparatus, where it recruits TANK-binding kinase 1 (TBK1) to phosphorylate the transcription factor interferon regulatory factor 3 (IRF3) and activating the “inhibitor of nuclear factor kappa-light-chain-enhancer of B cells (NF-κB)” (IKK) complex to release NF-κB (Figure 3). IRF3 and NFκB translocate to the nucleus and drive the synthesis of proinflammatory SASP genes, including interferons, inflammatory cytokines, and chemokines (Hopfner and Hornung, 2020; Decout et al., 2021). cGAS is essential for cellular senescence as its deletion abolishes the SASP and other markers of cellular senescence induced by either spontaneous immortalization or by DNA damaging agents (Yang et al., 2017). Furthermore, genetic engineering of mice to induce cGAS in microglia led to increased numbers of activated microglia, increased expression of type 1 interferon genes and inflammatory genes, and increased neurodegeneration (Gulen et al., 2023). Likewise, further work showed that inhibition of STING by use of a small molecule inhibitor abrogated the inflammatory response of senescent cells and attenuated age-related inflammation in multiple tissues including the brain (Gulen et al., 2023).

With respect to TBI, since DNA damage in the brain has been documented as early as 30 min and extending out to days after TBI, it would be expected that the cytosolic DNA sensing pathway, cGAS-STING would be similarly induced after TBI. Indeed, studies in male mice using the CCI model of TBI found that mitochondrial DNA was present in the cytosol of the ipsilateral cortex at 2 h after TBI (Fritsch et al., 2022), which corresponded with a significantly increased gene expression of STING in the ipsilateral cortex at 2 h, 4 h and 24 h after TBI (Fritsch et al., 2022). cGAS expression has also been reported to be significantly elevated in the injured mouse cortex at 24 h and 72 h after TBI, and this effect was significantly increased in aged mice (Abdullah et al., 2018; Barrett et al., 2020; Barrett et al., 2021). Although not examined in these studies, it would be interesting to determine whether cGAS expression in the injured cortex changes at earlier times after TBI, similar to that reported for STING expression (Fritsch et al., 2022). Corresponding to cGAS-STING pathway induction, expression of type 1 interferon response genes, which are downstream of STING in the cGAS-STING pathway, are significantly elevated in the mouse cortex at 2 h, 24 h, and 72 h after TBI (Barrett et al., 2020; Fritsch et al., 2022). Additional work has shown that upregulation of the type 1 interferon response plays a role in neurodegeneration and functional outcome after TBI, as interferon-beta (IFN-β) knockout mice had reduced neuroinflammation and neurodegeneration, as well as improved cognitive motor and cognitive outcome after TBI (Barrett et al., 2020). Interestingly, increased expression of STING has also been documented to occur in humans following TBI, as evidenced by significantly increased expression of STING mRNA in the human postmortem brain at 6 h after TBI (Abdullah et al., 2018). It should be noted that multiple cell types in the brain have been reported to show induction of cGAS or STING after TBI. For instance, Abdullah et al. (2018) reported that STING immunoreactive protein was significantly induced in astrocytes and neurons at 24 h after TBI in male mice. This study did not examine STING expression in microglia; however, another group that examined both male and female mice found that STING gene and protein expression was induced in microglia at 7 days after TBI (Packer et al., 2025). Likewise, in a separate study, cGAS gene expression was found to be significantly induced in microglia at 24 h after TBI in male mice (Fritsch et al., 2022).

To further determine the role of the cGAS-STING pathway in the neuropathology and functional outcome following TBI, several studies have used global, tissue-specific or cell-specific STING knockout (KO) mice, as well as global cGAS KO mice (Abdullah et al., 2018; Fritsch et al., 2022; Fritsch et al., 2024; Packer et al., 2024; Packer et al., 2025). These studies suggest that the cGAS-STING signaling pathway may play a key role in microglial activation, neuroinflammation, interferon response, neuronal injury and lesion volume, as well as motor and cognitive functional outcome after TBI (Abdullah et al., 2018; Fritsch et al., 2022; Fritsch et al., 2024; Packer et al., 2024; Packer et al., 2025). For instance, knockout of STING in either the brain or in the periphery (e.g., hematopoietic cells) of mice leads to smaller lesion size after TBI (Fritsch et al., 2024), which suggests that STING signaling in both the brain and in the periphery contributes to brain tissue loss after TBI. Further work has revealed that, in addition to its rapid activation in the brain, STING is also rapidly activated in white blood cells (WBCs) - as early as 2 h after TBI in male mice (Fritsch et al., 2024). The rapid STING activation in WBCs has been suggested to contribute to the early neuroinflammatory response after brain injury, which further promotes the influx of peripheral immune cells into the injured brain area and ultimately contributes to increased lesion volume, cell death and poorer outcome (Fritsch et al., 2024). Additional studies revealed that both global cGAS KO mice and STING KO mice have significantly reduced lesion volume and apoptotic cells in the cortex at 24 h and 14 days after TBI, further attesting to the role of the cGAS-STING pathway in contributing to enhanced lesion volume and cell death after TBI (Fritsch et al., 2022).

The beneficial effects on lesion size and cell death in cGAS and STING KO mice after TBI are likely due to reduced neuroinflammation, as both cGAS and STING KO mice had significantly decreased proinflammatory gene induction in the cortex at 24 h after TBI (Fritsch et al., 2022). Other studies using global STING KO mice also found that STING KO male and female mice had reduced microglial activation in the injured cortex, as well as decreased inflammatory and interferon-related gene expression, and improved cognitive outcome after TBI (Abdullah et al., 2018; Packer et al., 2024; Packer et al., 2025). These results were further confirmed through an alternate approach of administering the STING antagonist, chloroquine, which was found to similarly reverse cognitive deficits and reduce TBI-induced cortical inflammatory gene expression in male mice (Packer et al., 2024). Finally, studies using cell-specific STING KO mice have further revealed a key role for STING induction in microglia in neuroinflammation, cell death and functional deficits after TBI (Fritsch et al., 2024; Packer et al., 2025). For instance, Fritsch et al. (2024) found that microglia-specific STING KO mice had reduced TBI-induced lesion size, cell death and induction of the proinflammatory SASP factor, IL-1β in the injured cortex, as well as improved motor function after TBI. Likewise, Packer et al. (2025) reported that microglia-specific STING KO mice had decreased neuronal injury, interferon responses, and cortical inflammation after TBI, as well as reduced cognitive deficits. Table 1 summarizes the temporal activation and various roles implicated for the cGAS-STING pathway following TBI. Mechanistically, these studies provide evidence that cGAS aids in detecting injury-induced DNA damage to produce the second messenger cGAMP, which binds to and activates STING following TBI. It is suggested that activated STING signaling may help promote neuroinflammation after TBI by facilitating translocation of the transcription factors IRF3 and NFκB to the nucleus to help drive the synthesis of proinflammatory SASP genes. Additional studies are needed to further explore and extend these interesting findings.

**Table 1:** STING pathway regulation and potential role after TBI.

Time (post TBI)	Model	Key findings	References
2 h	Mice (CCI)	↑ mitochondrial DNA in cytosol, ↑ STING mRNA in cortex and WBCs, ↑ TNFα, IL-1β,type 1 IFN genes, ↓ lesion size in STING brain-specific or peripheral KO	Abdullah et al. (2018); Fritsch et al. (2022); Fritsch et al. (2024)
4 h	Mice (CCI)	↑ STING mRNA levels in cortex	Fritsch et al. (2022)
24 h	Mice (CCI)	↑ DNA damage in cortex, ↑ STING in neurons and astrocytes, ↑ SASP factors TNFα and IL-1β; ↑ astrocyte activation and microglial activation; all ↓ in STING KO and decreased lesion size in STING KO	Abdullah et al. (2018); Barrett et al. (2021); Fritsch et al. (2022)
24 h	Mice (CCI)	↑ cGAS in microglia; cGAS KO: ↓ lesion volume, ↓ pro-inflammatory genes, ↓ motor deficits	Fritsch et al. (2022)
24 h	Mice (CCI)	Microglia-specific STING KO had ↓ lesion size and ↓ apoptosis in the cortex	Fritsch et al. (2024)
72 h	Mice (CCI)	↑ cGAS, ↑ STING, ↑ IFN-β and other IFN-related genes in cortex; IFN-β KO: ↓ microglia activation and improved cognitive and motor function	Barrett et al. (2020)
4d	Mice (CCI)	Microglia-specific STING KO had ↓ motor deficits	Fritsch et al. (2024)
7d	Mice (FPI)	STING KO: microglial activation and inflammatory and IFN-related gene expression in cortex	Packer et al. (2024)
7d	Mice (FPI)	WT: ↑ STING expression in microglia, ↑ neuronal injury, ↑ IFN response, ↑ cortical inflammation; Microglial-specific STING KO: all these responses were attenuated, and improved cognitive outcome. Used both male and female mice	Packer et al. (2025)
14d, 21d, 28d	Mice (CCI)	IFN-β KO: ↓ long-term microglial activation and improved long-term motor and cognitive outcomes after TBI.	Barrett et al. (2020)

### Senolytic therapy and TBI

Senolytic drugs are compounds that selectively ablate senescent cells by inducing apoptosis (Figure 1). They were first identified based on the observation that senescent cells are resistant to apoptosis - due to up-regulation of pro-survival pathways termed SCAPs (Kirkland and Tchkonia, 2017). Identified SCAPs include BCL-2/BCL-xL, PI3K-Akt, p53/p21/serpines, HIF-1α, and dependence receptors/tyrosine kinases. The first senolytic drugs, dasatinib (D), a tyrosine kinase inhibitor, and the flavonoid, quercetin (Q), were shown to induce apoptosis in senescent cells, but not in non-senescent cells of adipose tissue, aorta, lungs and brain (Roos et al., 2016; Bussian et al., 2018; Justice et al., 2019; Wang et al., 2023). D reportedly targets the dependence receptors/tyrosine kinase SCAP and Q targets the BCL-2/BCL-XL, p53/p21/serpine, and PI3k/Akt SCAPs (Kirkland and Tchkonia, 2017). Other identified senolytic drugs include navitoclax (ABT 263), which targets the BCL-2 pathway (Zhu et al., 2016), and the BCL-XL inhibitors such as A1331852 and A1155463, and the flavonoid, fisetin, which targets the BCL-2, p53/p21/serpines, and PI3k/Akt SCAPs (Zhu et al., 2017). Interestingly, D + Q is an FDA approved drug/dietary supplement, which has been shown in human studies to be well-tolerated and to reduce senescent cells in various tissues of the body (Hickson et al., 2019; Justice et al., 2019). D + Q has also been shown to ablate senescent cells in the brain of an Aβ mouse model, reduce neuroinflammation and Aβ load, and reduce cognitive deficits (Zhang et al., 2019). In addition to senolytic drugs, senomorphic drugs are compounds that target pathways involved in SASP production to suppress harmful SASP effects without killing senescent cells. Putative senomorphic agents include mTOR inhibitors, JAK/STAT inhibitors, HDAC inhibitors, p38 MAPK inhibitors, and IKK-NFκβ inhibitors (Kirkland and Tchkonia, 2020; Zhang et al., 2021; Saliev and Singh, 2025). However, most studies focus on using senolytic drugs over senomorphic drugs as senolytic drugs allow intermittent “hit and run” dosing that allows clearing out of senescent cells to prevent spread of injury and allow repair, while senomorphic drugs require continuous life-long administration to suppress harmful cell signals and products.

As shown in Table 2, a number of studies have examined the effects of several purported senolytic drugs in preclinical and clinical TBI. To assist the reader in understanding this body of literature, the doses, routes and timing of the senolytic drugs used in each study, the species and TBI model used, as well as the major findings and strengths/limitations of each study are listed in Table 2. It should be noted that many of the studies utilized drugs that only later were discovered to have senolytic properties – such as curcumin, fisetin, dasatinib and quercetin. Thus, unfortunately, effects on cellular senescence and the SASP were not examined in these studies. In such cases, we have noted this under limitations listed for that study in Table 2. However, the beneficial effects of these drugs on many other molecular and functional endpoints were documented, as outlined under the major findings in Table 2. Other studies, using classical senolytic drugs such as D + Q and ABT 263, did examine the effects upon cellular senescence and the SASP, and are discussed in more detail below.

**Table 2:** Senolytic drug treatments after pre-clinical or clinical TBI.

Drug	Dose/Route/Timing	Species	TBI model	Main findings	Strengths (S) Limitations (L)	References
Pre-clinical						
Dasatinib + Quercetin	D:5 mg/kg/day Q 50 mg/kg/day Orally beginning 1 month post-injury, 3 days/ week for 13 weeks	Mice	Controlled cortical impact	Improved cognitive and depressive-like behavior, reduced neurodegeneration and neuron loss, reduced p16-and p21-positive glial cells and SASP factors IL-1β and IL-6 in the brain	(S) Studied multiple markers of cell senescence and functional endpoints, showed a prolonged therapeutic window for D + Q (L) Only males were studied	Wang et al. (2023)
Dasatinib	25 mg/kg, i.p., daily beginning at 2 h after TBI	Mice	Moderate lateral fluid percussion	Improved cognitive and motor outcomes, reduced inflammation and neurodegeneration	(S) Studied multiple molecular and functional endpoints (L) Only males were studied. Did not examine cellular senescence	Yu et al. (2016)
Navitoclax (ABT-263)	1.5 mg/kg, i.p., 1 week post-injury.	Mice	Repeated mild (controlled cortical impact) closed head injury	Enhanced cognitive function in males 2 weeks post-injury, sex-specific effect of ABT-263 to reduce injury-induced expression of senescence markers in males, but not in females	(S) Examined cellular senescence in both males and females (L) Only males were examined in behavioral studies.	Schwab et al. (2022)
Quercetin	30 mg/kg i.p., daily for 3 days post-injury	Rats	Weight drop	Improved cognitive function, improved antioxidant enzymes expression, reduced cell death	(S) Studied female rats and both molecular and cognitive endpoints (L) Did not examine cellular senescence or male animals	Yang et al. (2014)
Quercetin	25 μmol/kg, i.p., 1 h post-injury	Rats	Moderate anterior midline fluid percussion	Improved compound action potential amplitudes, reduced oxidative stress	(S) Examined multiple endpoints 1–3 days after TBI (L) Only studied males, did not examine cellular senescence or behavioral outcomes.	Schultke et al. (2005)
Quercetin	50 mg/kg, i.p., 0.5 h post-injury	Mice	Weight drop	Improved mitochondrial function and antioxidant defense at 24 h after TBI	(S) Showed beneficial effects upon mitochondrial biogenesis (L) Only studied males, did not examine cellular senescence or behavioral outcomes.	Li et al. (2016)
Quercetin	50 mg/kg, i.p., 0.5 h, 12 h, 24 h post-injury	Rats	Weight drop	Reduced neuronal autophagy and apoptosis, enhanced pro-survival PI3K-Akt signaling in neurons, improved motor and cognitive function	(S) Studied multiple molecular and functional endpoints (L) Only studied males, did not examine cellular senescence	Du et al. (2016)
Fisetin	25, 50 or 75 mg/kg, i.p., 0.5 h post-injury	Mice	Weight drop	Improved motor function, reduced oxidative stress, decreased lesion volume	(S) Studied multiple molecular endpoints and motor function (L) Only studied males, did not examine cellular senescence or cognitive outcome	Zhang et al. (2018)
Curcumin	500 ppm, orally 1 month pre-injury	Rats	Mild fluid percussion injury	Decreased microglial/macrophage activation, reduced neuronal apoptosis	(S) Curcumin supplementation was in the diet. Examined multiple endpoints (L) Only studied males, did not examine cellular senescence	Wu et al. (2006)
Curcumin	30 mg/kg/day i.p. for 4 weeks after TBI	Rats	Controlled cortical impact	Improved cognitive outcome, reduced chronic inflammation, enhanced neurogenesis	(S) Examined multiple endpoints long-term (28 days) after TBI (L) Only studied males, did not examine cellular senescence	Sun et al. (2020)
Curcumin	100 mg/kg, i.p., 5d pre-injury	Rats	Weight drop	Improved motor function, reduced brain injury, reduced oxidative stress	(S) Examined multiple endpoints 2 weeks after TBI (L) Only studied males, did not examine cellular senescence or cognitive function	Samini et al. (2013)
Clinical						
Curcumin	500 mg, via enteral nutrition, daily for 7d	Human	ICU-admitted adult TBI patients	Improved nutritional status and reduced serum levels of IL-6, TNF-alpha, MCP-1 and C-reactive protein	(S) Randomized study with homogeneous population (L) Length of intervention was short and did not examine cognitive function or long-term outcome	Zahedi et al. (2021)

#### Preclinical studies

The first report on the effectiveness of senolytic drug ablation of senescent cells in the injured brain after TBI was a study that examined the effect of the senolytic drug navitoclax (ABT 263) in a repeated mild TBI mouse model (Schwab et al., 2022). In the study, ABT 263 was administered by a single intraperitoneal injection 1 week after the last mild repeated TBI (Table 2). Examination of the mice 1 week after ABT 263 treatment revealed that the senolytic treatment significantly reduced cellular senescence in the cortex and hippocampus of male mice after repeated mild TBI as compared to vehicle controls, as evidenced by reduced p21 protein levels (Schwab et al., 2022). Female mice also showed a similar trend for reduced cellular senescence, which however was not statistically significant. Similarly, cGAS-STING signaling exhibited a trend for reduction by ABT 263 treatment in males and female mice after repeated mild TBI, but the effect did not reach statistical significance. Finally, using the Morris water maze, the investigators showed that ABT 263 treatment significantly improved spatial memory and executive function after repeated mild TBI in male mice (Schwab et al., 2022). However, female mice were not examined. It is unclear why there was a sex difference in the effect of ABT 263 on cellular senescence following repeated mild TBI in mice. The authors did note that the senescent markers had stabilized in female TBI mice by the 2 weeks post-injury timepoint used in the study, while they were still elevated in male mice, and thus the lack of reduction of these senescent markers by ABT 263 in female mice could be due this lack of continued elevation. In addition, it would be interesting to examine a longer treatment with ABT 263 as another study showed reduced cellular senescence and improved cognitive function in females in an AD mouse model after a longer intermittent treatment period for several months with the senolytic cocktail D + Q (Fang et al., 2025). Interestingly, an additional study on repeated mild TBI in male rats found that the sex hormone, testosterone was significantly decreased in the serum of the male rats at 4 weeks after the last mild TBI (Exline et al., 2025). The authors noted that the decrease in testosterone levels was associated with persistent memory defects and an increase in the cellular senescence markers, p16, p21 and p53 in the hippocampus at 9 weeks after the last mild TBI, suggesting a potential association between the levels of testosterone and degree of cellular senescence and cognitive function defects after TBI. Furthermore, testosterone administration initiated at 5 weeks after the last mild TBI attenuated cellular senescence and ameliorated the persistent memory defects. While testosterone is generally not considered a classical senolytic compound, there are number of reports of it reducing cellular senescence markers and SASP factors in a variety of cells (Ng and Hazrati, 2022). The mechanisms underlying testosterone’s effects are currently poorly understood, but testosterone is well known to exhibit potent anti-inflammatory effects, which may contribute to these observed effects. Future studies are needed to better clarify the effects of gender and sex steroids on cellular senescence, as well as the effects of senolytic drugs after TBI.

As also indicated in Table 2, our group also recently used an intermittent D + Q treatment to examine its effectiveness in ablating senescent cells and its potential beneficial effects on long-term cognitive outcome in male mice following CCI (Wang et al., 2023). In the study, D + Q was administered orally by gavage beginning 1 month after TBI for 3 consecutive days per week for 13 weeks. The study revealed that the senescent cell markers, p16 and p21 were co-localized in astrocytes and microglia of male mice at 5 weeks post injury and 4 months post injury in various brain regions, and that intermittent administration of the senolytic drugs, significantly ablated the senescent cells, as well as significantly reduced expression of the SASP pro-inflammatory factors, IL-1β and IL-6. D + Q also significantly attenuated neurodegeneration and significantly rescued defects in spatial reference memory and recognition memory, as well as depression-like behavior in TBI mice (Wang et al., 2023). The results suggest that an extended therapeutic window may exist for beneficial effects of senolytic therapy in TBI. Since our study administered D + Q orally, we cannot rule out that its effects could be mediated peripherally. However, previous studies have demonstrated that D and Q can cross the blood brain barrier (Porkka et al., 2008; Ishisaka et al., 2011; Zhang et al., 2019), and thus a central mechanism of action is also possible. Additional studies are needed to address this issue. From a translational perspective, an oral route for senolytic drug administration offers several potential advantages, such as (1) non-invasiveness, (2) convenience of drug administration, and (3) better patient compliance. Furthermore, intermittent dosing with senolytics has the potential advantage that it may minimize the chance of adverse side effects of the drugs. Indeed, intermittent D + Q administration has already been shown to be well tolerated, safe, and effective in reducing senescent cell burden in humans (Hickson et al., 2019; Nambiar et al., 2023; Garbarino et al., 2025). However, it should be noted that a recent study in mice reported that intermittent D + Q administration decreased oligodendrocyte function and myelination in the corpus callosum (Lombardo et al., 2026). Thus, one cannot completely rule out the potential for unintended negative side effects of senolytics, and additional studies are needed to confirm their safety and specificity. Table 2 also shows that dasatinib and quercetin administered alone were also beneficial in TBI (Schultke et al., 2005; Yang et al., 2014; Du et al., 2016; Li et al., 2016; Yu et al., 2016). However, unfortunately, the studies did not examine effects on cell senescence or SASP. Likewise, fisetin, which recently was demonstrated to be a highly potent senolytic compound (Yousefzadeh et al., 2018), and curcumin, a plant-based polyphenol found in turmeric spice with potent anti-inflammatory properties (Luis et al., 2022), have both been shown to exert beneficial effects in TBI (Zhang et al., 2018). Unfortunately, these studies also did not examine cell senescence or SASP. Hence, future studies are needed to examine the senolytic ability of fisetin and curcumin, as well as dasatinib and quercetin administered alone, after TBI.

Finally, there should be a word of caution that demonstration of ablation of cells by senolytic drugs in these preclinical studies does not in itself prove causation (e.g., that the beneficial effects of the drugs are solely or primarily due to their senolytic ability). This is especially true as the drugs can affect many pathways which could alternatively explain their beneficial effects. Future studies utilizing non-pharmacological approaches such as genetic ablation of senescent cells could help address this issue and further confirm the role of cell senescence in TBI pathology and outcome. Genetic ablation of senescent cells could be accomplished by targeting p16-positive cells via a transgene system like the INK-ATTAC or p16-3 MR system. This genetic approach has already been used successfully in previous studies to reverse age-related tissue dysfunctions and extend the lifespan in mice (Baker et al., 2011; Beck et al., 2020; Mahoney et al., 2024), as well as to reduce tau aggregation and neurodegeneration in mouse models of AD (Bussian et al., 2018). Thus, similar genetic studies in TBI could help advance our understanding on the role of cellular senescence in TBI.

#### Clinical studies

Finally, to our knowledge, only one study has examined the potential beneficial effects of a senolytic drug in clinical TBI – by examining the senolytic drug curcumin in intensive care unit (ICU)-admitted adult TBI patients (Zahedi et al., 2020). In the study, placebo or curcumin C3 complex (a highly enriched turmeric extract standardized to 95% curcuminoids) was administered with piperine (main ingredient in black pepper that dramatically boosts curcumin bioavailability) enterally at a dose of 500 mg for 7 days to intensive care unit (ICU)-admitted adult TBI patients (Table 2). The study revealed that curcumin C3 complex significantly reduced serum SASP factors, IL-6, TNF-*α* and Chemokine (C-C motif) ligand 2 (CCL2), as well as C-reactive protein, a systemic marker used to measure downstream effects of SASP activity in the body. In addition, curcumin C3 complex treatment significantly improved scores for tests that measured overall disease severity, as well as risks of malnutrition and death.

#### Therapeutic implications – caveats, potential limitations, and future directions

The above described preclinical and clinical studies show potentially promising beneficial effects for senolytic drugs after TBI. However there are still a number of barriers that must be overcome before clinical translation in TBI can be achieved. These include: (1) better determination of the optimal timing of administration and doses for senolytic drugs, (2) more studies to carefully establish blood brain barrier penetrability and pharmacodynamics for the drugs in both animals and humans, (3) careful consideration and evaluation of injury heterogeneity of TBI, the potential systemic effects of the drugs, and safety in acutely injured patients, (4) the need for more studies in females due to the preponderance of studies so far being in males, (5) determination of whether senolytic drugs may cause impairment of acute repair processes, and (6) once justified and better informed by more preclinical studies, TBI-specific human trials could be conducted to test safety and efficacy of senolytic drugs (and potentially senomorphic drugs) in the clinical situation.

## Summary and conclusions

In summary, these findings demonstrate that cellular senescence is induced acutely and chronically in the brain after either a single moderate to severe TBI or repeated mild TBI, which leads to SASP production that may contribute to neuroinflammation and neurodegeneration in the injured brain. Knockout and antagonist studies further indicate that the cGAS-STING pathway plays an important role in detecting DNA in the cytosol of damaged cells in the injured brain, and connecting cellular damage to SASP production. Senolytic therapy in animal models reduced lesion size, inhibited neuroinflammation, attenuated neurodegeneration, and enhanced functional outcome when administered acutely or long-term after TBI. While collectively these findings are suggestive of a potential role of cellular senescence in neuroinflammation, neurodegeneration and outcome after TBI, additional studies are needed to further confirm these results, and provide better understanding and firmer conclusions on the role of cellular senescence in TBI. At this point, it should be noted that clinical applications of senolytic therapy remain investigational, and require significant more study before potential translation to the clinic. In closing, while much has been discovered concerning cellular senescence and TBI, much remains unknown, and more studies are needed to expand our knowledge in this important area and to further explore the potential therapeutic efficacy of targeting this key mechanism.

## Glossary

AD: Alzheimer’s disease
BCL-2: B cell lymphoma-2
Bak: B cell lymphoma-2 antagonist/killer
BAX: B cell lymphoma-2 associated X, Apoptosis Regulator
BCL-xL: B cell lymphoma-extra-large
cGAS: cGAS-STING (cyclic GMP-AMP synthase)
STING: stimulator of interferon genes
CCI: controlled cortical impact
CCL2: Chemokine (C-C motif) ligand 2
DDR: Dasatinib (D), DNA damage response
HDAC: Histone Deacetylase
HIF-1α: Hypoxia inducible factor 1 subunit alpha
IKK: inhibitor of nuclear factor kappa-light-chain-enhancer of B cells
IFN: Interferon
IL: interleukin
IRF3: transcription factor interferon regulatory factor 3
JAK/STAT: Janus kinase/signal transducers and activators of transcription
KO: Knockout
mTOR: mammalian target of rapamycin
MAPKs: mitogen-activated protein kinases
MCP-1: monocyte chemoattractant protein-1
NF-κB: Nuclear factor kappa-light-chain-enhancer of B cells
PD: Parkinson’s disease
PI3K: Phosphatidylinositol 3-kinase
PCNA: Proliferating cell nuclear antigen
ROS: quercetin (Q), reactive oxygen species
SA-β-gal: senescence-associated β-galactosidase
SASP: senescence associated secretory phenotype
SCAPs: senescent cell anti-apoptotic pathways
TBK1: TANK-binding kinase 1
TBI: traumatic brain injury
TNF-α: tumor necrosis factor-alpha
WBCs: white blood cells
WT: wild type
